# Towards an understanding of medical student resilience in longitudinal integrated clerkships

**DOI:** 10.1186/s12909-015-0404-4

**Published:** 2015-08-21

**Authors:** Jennene Greenhill, Ken R. Fielke, Janet N. Richards, Leesa J. Walker, Lucie K. Walters

**Affiliations:** Flinders University Rural Clinical School, South Australia, PO Box 852, Renmark, 5341 South Australia

**Keywords:** Longitudinal integrated clerkship, Resilience, Medical student, Medical education, Transformative learning theory, Agentic learning, Social learning theory, Interpretive approach

## Abstract

**Background:**

Resilience is required to succeed academically, overcome challenges during clinical training and cope positively with stress in future professional life. With medical students at high risk of mental illness, socially accountable medical schools are seeking to foster student resilience. This exploratory study proposes a conceptual framework for student resilience in longitudinal integrated clerkships (LICs).

**Methods:**

This qualitative study sought to understand student resilience during the first year of clinical training in a rural LIC where there were consistent anecdotal reports of high student resilience. In-depth interviews were conducted with a purposive sample of 19 medical students, professional staff and clinician teachers. An interpretive approach was used to analyse the data with emerging concepts compared to define evolving theoretical constructs, and develop a conceptual framework.

**Results:**

LIC students experienced adversity during the first clinical year of the medical course due to challenges encountered in the learning environment. This distress was moderated by: a secure, supportive learning environment; their profound learning journey; and utilisation of organisational structures to stay on course.

**Conclusion:**

This triad of inter-related themes forms a conceptual model that challenges simplistic notions that medical courses should focus solely on providing tangible and emotional supports for students. How LIC programs may contribute to student wellbeing is discussed through the lenses of agentic, reflective and transformative learning.

## Background

Resilience is defined as “a dynamic process wherein individuals display positive adaptation despite experiences of significant adversity or trauma” [1]. A longitudinal, multi-institutional study found that resilient medical students were less likely to experience depression than vulnerable medical students [2]. Resilient students also report greater wellbeing including: better quality of life, a positive outlook on their learning environment, and academic success [2, 3]. Medical students are at similar risk of depression, suicide and burnout when compared with other university students, and at higher risk than their aged matched peers in the general population [4, 5]. In Australia, many rural clinical schools (RCS) relocate medical students from metropolitan areas for clinical training, a particularly challenging part of the medical course [6, 7]. A recent Australian-wide Beyondblue study found that medical students on rural placement suffered higher levels of mental illness than metropolitan-based students [8].

Socially responsible medical schools have a responsibility to develop and foster student resilience during clinical learning [9]. This research was conducted in response to a number of serious incidents occurring with distressed medical students associated with the metropolitan block rotation program at an Australian university. This situation prompted academics to critically review the University’s rural Longitudinal Integrated Clerkship (LIC) program where student resilience seemed better supported.

Resilience is recognised as being domain specific [10, 11] and context specific [12, 13]. In this article we identify clinical resilience as the capacity to bounce back from the tensions associated with taking on a student-doctor role with the associated professional and knowledge expectations. Several medical schools have introduced sessions designed to promote resilience and wellbeing including: wellness models, mindfulness training, and behavioural-change strategies. These programmed sessions aim to decrease stigma and facilitate self-help and giving assistance to others [14–19]. However no agreement has been reached on which strategies are most effective or how they should be embedded within the curriculum. Anecdotally the continuity of relationships between the students and their clinicians, support staff and peers facilitated by the longitudinal program was thought to support student resilience. An important psychosocial factor associated with the development of resilience to a wide variety of stressors is social support [19, 20]. Social support refers to an individual’s ability to connect with others through new or existing relationships in times of stress, in order to access emotional, cohesional, informational or instrumental resources [7, 21]. This provided authors with an initial conceptual framework for this study.

The purpose of this exploratory study was to address this gap in the literature by developing a conceptual framework for student resilience in a LIC curriculum. This work will then allow the model to be tested in broader medical curriculum contexts.

Flinders University offers a four year Doctor of Medicine program, in which third year students can select a one year rural community-based LIC known as the Parallel Rural Community Curriculum (PRCC) [20]. Students are exposed to all medical disciplines simultaneously and continuity of supervision facilitates a progressive increase in responsibility [21]. Groups of eight to ten students are supported by administrators who manage accommodation, timetabling of clinical sessions and local teaching programs. Each PRCC has at least one practicing GP with a fractional academic appointment who provides: local leadership, mentorship and facilitated clinical learning opportunities [22].

## Methods

**Fig. 1:**
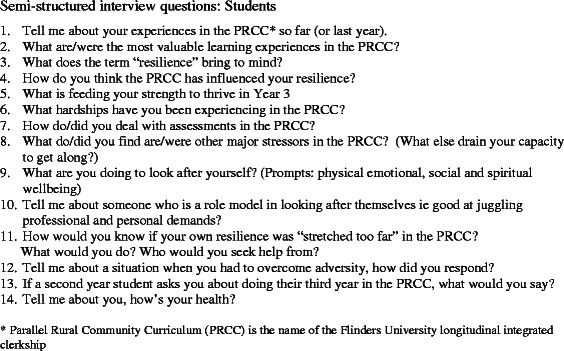
Semi-structured interview questions for students

**Fig. 2:**
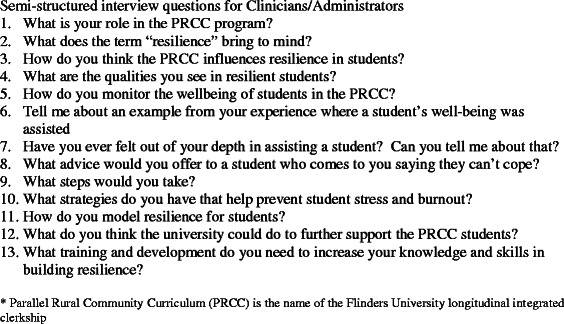
Semi-structured interview questions-Clinicians and Administrators

An interpretive approach was used to develop a conceptual framework for how a LIC program can develop and support student resilience [23]. A purposive sample of students, program administrators and clinician teachers were recruited from across four rural PRCC regions. Seeking current and retrospective perspectives, the student participants included current 3^rd^ year LIC students and 4^th^ years LIC alumni. Open ended questions explored the development of student resilience during a single, in-depth interview. The interview sensitively enquired about personal and academic difficulties students experienced, coping strategies they adopted and which aspects of the LIC program were supportive or challenging (Fig. 1). The program administrators and clinical teachers were asked open ended questions to explore their understanding of resilience and how aspects of the LIC program might influence student resilience (Fig. 2).

**Fig. 3:**
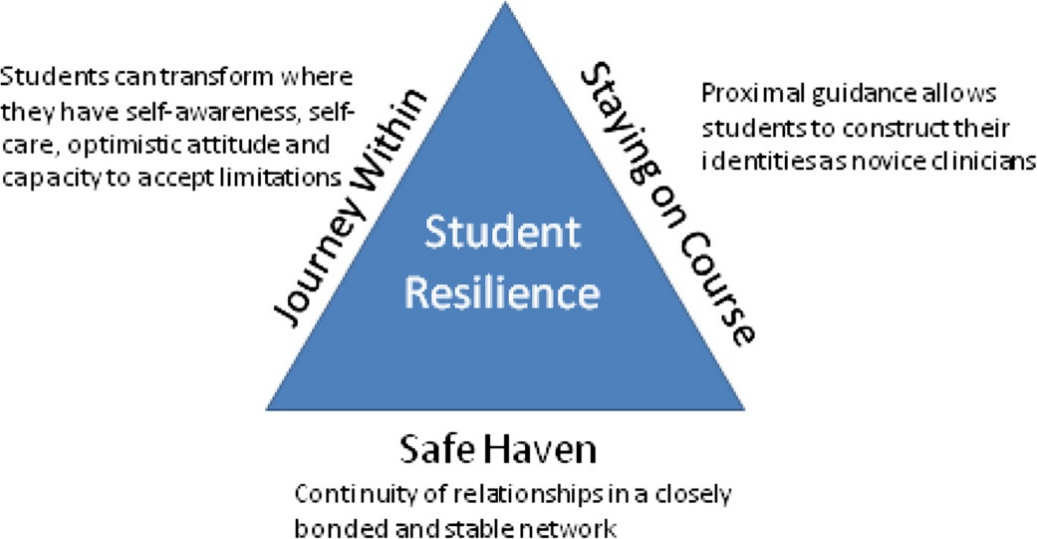
Flinders Model of Student Resilience in a LIC curriculum

All interviews were conducted toward the end of the academic year by one team member (KF) to provide consistency. A practising psychiatrist who taught the students as a clinical teacher, (KF) relinquished his assessment role in the year of the study to avoid any power differential that may exist between the student and interviewer. Interviews, averaging an hour in length, were recorded, transcribed, and de-identified prior to the initial open coding by two research team members (JR, JG). All authors performed selective coding to identify emergent themes until saturation was reached. An inductive process involving detailed discussion and constant comparison between researchers was undertaken to develop consensus around themes and link theoretical concepts. The two clinical teacher interviews were adequate to triangulate student and administrative staff data with no new themes emerging. An audit trail using nVivo9, member checking and triangulation of the data enhanced rigor. Participants gave written informed consent. Ethics approval was obtained from Flinders University Social and Behavioural Human Research Ethics Committee (5673).

## Results

Nineteen participants were recruited (Table 1) comprising: nine medical students, eight program administrators, and two clinical teachers. Four emergent themes contribute to an understanding of the development of resilience in the LIC: (1) Evidence of distress, (2) A Safe Haven, (3) Journey within and (4) Staying on course. Each theme was elucidated through subthemes

**Table 1:** Research participants

		Potential participants	Study participants	Participant labels
Students	Current LIC students	33	5	S3_1, S3_2, S3_3, S3_4, S3_5,
	Previous years LIC students	33	4	S4_1, S4_2, S4_3, S4_4,
Professional staff (administrative)		9	8	A1,A2,A3,A4,A5,A6,A7,A8
Clinician Teachers		^a^	2	CT1, CT2

^a^ Only two clinician teachers were approached to participate in this study as their voice was used solely to triangulate themes developed from students and professional staff

### Evidence of distress

Participants reported that all students experienced stress and some students became distressed due to the pressure they faced during their first clinical year, providing evidence that students are required to adapt to significant challenges (adversity) in order to succeed. High stakes exams were seen as the major cause of stress, however students also highlighted their new role as novice clinicians and relocation as stressful.

#### Apprehension

Students reported apprehension even in anticipation of the year.

*“…the myth of third year. People call it the Olympics, the worst year. You see the third years. You smell the third years by the end of it, and they just act like crazy. So I was feeling physically nauseous before the week started and I was getting irritable at home because I was visiting my parents and I said to them “I know I am uptight, I’ll be better once it starts” …Now…. I like being here.” S3_1.*

Participants reflected on ethical dilemmas and human suffering they were experiencing.

*Enough courage to get up every day and go in [laughs] to see what you’re going to face. I had one week where I got a bit down because I identified three people in ten days that had cancer and that was a bit shit. S3_1*

#### Pressure to achieve

Clinical teachers reported that strategic learners put considerable pressure on themselves to achieve.

*“I find the ones that we've had more trouble with have been those that have come down [to the LIC site] so focussed on getting an HD [high distinction] that everything else has just gone out the window and if something upsets the cart along the way it can be pretty stressful.” CT2*

As the year progressed the end of year barrier exam was the major focus. Stress levels heightened and fatigue set in.

*“…probably two weeks out from the exams …I do remember quite clearly driving home and just thinking about “if I just ran my car off the road into that tree, I wondered if it would hurt?”…I was reaching the end of my tether.S4_1*

#### Safe Haven

Participants described how support was provided during the LIC, encouraging students to fully explore different clinical contexts. Students could return to ‘home base’ (the rural clinical school office in their PRCC region) to debrief and rejuvenate when needed. Staff described getting to know the students individually over time. With small cohorts of students forming close relationships, peer-support occurred. Students reported sharing their learning journey with peers, staff and clinicians and therefore they did not feel alone. This safe haven provided students with emotional support, guidance and a sense of belonging and was described as a foundation for the LIC learning journey.

#### Culture of respect

Administrators recognised the student cohorts vary in age, gender and cultural origins. The staff and clinical teachers reported actively working to establish a deep culture of mutual support, openness, equity, respect and trust with their student groups.

*“You’ve got to realise that not everybody’s the same. We’ve got different cultural backgrounds, different family backgrounds …you just have to have that respect”.A7*

#### Knowing the students

Students reported that continuity with clinical teachers and administrators provided a sense of stability. They felt well supported by administrators who came to know them individually:

*“…she’s [program administrator] been somebody that’s been a constant, which has been really lovely, because I feel that she knows me….she just helps”. S3_1*

Administrators provided pastoral care, and liaison between students and the academic and clinical staff. There was a strong focus by administrative staff on student physical and emotional support. They described consciously monitoring wellbeing and anticipating when students may struggle.

*“we [administrative staff] always encourage the students to come to us. I feel like they have approached me on occasions when they’re anxious and where I can I’ve taken them out for coffee and discussions, given them ideas about how they may deal with it and whether it’s talking to their clinical educator, academics or family. … I guess there is some times where they’re not feeling so chipper, but it’s not visible. You can hear them laughing and you think everything’s fine and as I’ve often heard, you can hear teaching going on and heaps of laughter and then at the end when everyone’s going you’ve got one left behind crying “A5*

In contrast, a few students reported some tension related to the familiar relationships they had with University staff in local sites. One student described disclosing to a clinical supervisor that s/he was struggling with isolation. The student suspected the information was passed from a clinical supervisor to the university. Students reported these blurred boundaries between informal support and formal responsibility to the university could occasionally reduce their comfort with accessing staff support.

#### Peer group support

The orientation program proactively provided opportunities to develop positive peer relationships which enhance group interaction throughout the year, as this student comments:

*“We had not a lot of contact because we had such different friendship groups ….you kind of pull together in orientation week because you’ve all relocated from the city, so you’re in this together, so that kind of got us all talking” S3_1.*

Establishing constructive peer dynamics was important for maintaining a sense of security within the group. Students reported developing a sense of team-cohesion as they studied together, supported each other and socialised.

*“having everybody with a common goal and a common method of managing those problems really helps because I feel like I am part of a team…” S4_2*

However, it was recognised that the stress of looming exams could undo this team-cohesion as people prioritised their own needs making trusting relationships with staff and academics vital.

*“as the pressure builds towards the end of year exams cracks appear in the group and stress is amplified”.CT2*

The safe haven theme represents the strong perceptions students have of assistance being available to them while they adapt to the stressors of clinical work and looming exams. These supportive resources were demonstrated to include: emotional (such as nurturing), informational, companionship, and intangible (such as personal advice).

#### The Journey Within

Participants identified that students developed or drew upon positive coping strategies including: being self-aware, practising self-care, optimism, help-seeking behaviour and accepting personal limitations.

#### Self-awareness

According to students, self-awareness was important for monitoring their own wellbeing. Some students described previous experiences with adversity and demonstrated the maturity to draw upon their life experiences. Students identified a need to seek assistance early.

*“…I sent her [academic coordinator] an email going, I think I’m at the bottom bit of the dip in the graph that you showed us and I know I am there because all of these other things have just seemed way too hard, and way overwhelming” S3_3*

#### Self-Care

Staying healthy and making time to relax when feeling stressed was reported as important. Self-care strategies described included enough sleep, eating well, avoiding alcohol and drugs, exercising, listening to music, meditating, visiting family, debriefing with peers, socialising or just getting outdoors.

*“In those situations I think I just need to ride the depression out… get outside a bit more often, get a bit more sunlight, more physical activity.” S4_4*

Students reported being advised to find their own doctor and being provided with access to independent counselling services. Self-care was also reported to involve self-compassion.

*“… giving yourself permission to feel that way. And that it’s a period of three or four days, or whatever, over a longer period of time, where you just feel like this is all gone to wrack and ruin” S3_3*

Taking time to enjoy nature and other more spiritual pursuits were described as ways to realize a more balanced life.

#### Optimistic attitude

Students reported ongoing, incremental learning in the clinical setting: “you’re doing it, purely through exposure”. They reported feeling more resilient when they were able to “be flexible” and “laid back’ and ‘enjoy the ride’. Participants recognised that the learning journey was challenging and “it is OK to struggle” but students were determined to succeed in their meaningful career goals.

#### Accepting limitations

Participants described more resilient students were able to be “realistic about what’s achievable”. One student clearly acknowledged personal limitations:

*“I guess I see strength there in just about knowing my own limitations and when to ask for help too, which is I think something that people and medical students and doctors generally are not very good at is asking for help. S4_2*

The theme ‘journey within’ recognises that resilience was a dynamic internal dialogue whereby students can build on their personal attributes and developed new strategies to reframe and cope with clinical experiences and exam pressures.

#### Staying on course

Participants reported the importance of the LICs organisational structures in providing students with educational supports. Students received coherent advice on navigating the LIC curriculum reinforced by the continuity of supervision from clinical supervisors who were invested in their success. Wellbeing sessions in stress management and self-care were included in the formal curriculum. A clear protocol was established for students to confidentially seek help and for staff and faculty to assisting students in distress.

#### Curriculum compass

Academics shared their perspective on their learning journey. Academic and clinical staff prepared students for the challenging times ahead and reinforced the way LIC students would experience a learning journey differently from block rotations.

*“part of our O (orientation) week, we have the academic talk about those peaks and troughs [observed emotional highs and lows students experience at identified times during the LIC academic year] and how you're going to feel. But we feel that we need to expand on that” A1*

Some students perceived the LIC curriculum to be unstructured in comparison to the discipline based block rotation. Clinical teachers described the students’ adjustment to an integrated curriculum as challenging until they became familiar with the learning milestones. Timetabled teaching and clinical activities reportedly provided opportunities for frequent feedback enabling students and their clinical supervisors to adjust their learning goals as needed. These structures were not initially recognised and appreciated by students as systematic.

*“…we have this system where you have to pick it up over the whole 12 months and they tell you along the way "just have faith, the system works" and it does, you do get through it, but at the time it causes you so much stress …” S4_1*

#### Expert guides

When clinical teachers and local academics were strongly engaged, their expert guidance was recognised and valued by students:

*I think it’s important that you’ve got somebody [GP supervisor] who’s more experienced than you, who’s got more runs on the board than you have, where you can go to them and go, “I saw this. What should I do with it? Should I do anything with it? How should I actually approach this?”S3_3*

Participants reported that clinical teachers encouraged students to participate actively in clinical environments while ensuring they were safe, and learned from mistakes.

*“…You learn in an environment where you feel it is alright to make errors and mistakes and make a bit of a fool of yourself and if you are afraid of doing that, then I don’t think you learn to your best capacity …..I think by putting yourself in situations in the medical course where you are confronted by your own limitations and lack of knowledge you can learn a great deal.” S4_2*

Local academics reported continually monitoring students through multisource feedback.

*“I try and sit down with them or have a phone conversation periodically through the year; on just pretty much how things are going. More formally through the faculty meetings… we have a discussion about each student then – and I rely on the staff … they get a good feeling of whose not coping or whose having a lot of issues… and follow up on that.”CT1*

#### Plans for troubled waters

Participants described the formal wellbeing program, which provided sessions on self-care early in the year and stress management activities near the end of the year as beneficial. When problems were identified, participants reported that students were assisted to get ‘back on course’ through: local counselling services, sick leave and negotiated learning plans, before instituting formal remediation.

*“Put in supports if required. So giving you a couple of days off, not without question. I think if you’re that bad other people are going to have noticed anyway so, well, with the student here that required time off everyone knew and it was kind of - then they realised as well after we’ve spoken to them and it was quite easy for her to get time off. Then if she wanted they put in place other things like psychology or whatever. So I think they do quite a lot for the students’ health and wellbeing out here” S3_5*

## Discussion

LIC students do experience significant challenges (adversity) in the clinical learning environment. Adversity is recognised in the literature as a prerequisite for developing resilience in any domain [24, 25]. Clinical demands and examinations were cited as the major sources of stress for LIC medical students which is consistent with other Australian studies [19, 26]. Beyondblue found high levels of distress and psychiatric disorders persist from medical school into junior doctor years, indicating a culture of suffering early in medical careers [27].

The Flinders model of student resilience constitutes a triad of inter-dependant themes: a safe haven, the journey within and staying on course (Fig. 3). The *safe haven* underpins the student journey by providing an inclusive, strong support network for students. Students are able to maintain resilience when provided with the resources to *stay on course* as a result of the continuity of expert supervision and guidance offered by the LIC staff and educators. The *journey within* describes how students draw on and develop positive coping strategies [28, 29] and reaffirms the meaningfulness of their goal to become a doctor. The three themes outlined in this Flinders conceptual framework of resilience align with Antonovsky’s Salutogenic model which proposes that three qualities contribute to a *sense of coherence* [30]. These are: meaningfulness (which aligns with the journey within); comprehensibility (which has similarities to staying on course); and manageability (which parallels the safe haven) [30].

A *safe haven* is integral to an effective learning environment in the LIC program. In this study medical student resilience is enhanced by having access to a safe haven with closely bonded and stable interpersonal networks [31]. Continuity of relationships in LICs provides capacity to secure students’ physical and emotional wellbeing. A safe haven was demonstrated to act as a ‘secure base’ allowing students confidence to push their limits and return for solace as necessary [32].

Medical school is a time of significant psychological distress [33]. Vulnerable students have previously been considered at greater risk during this time [5]. However, the theme *the journey within* illustrates that, independent of other personality characteristics, attributes including self-awareness, self-care and accepting limitations are recognised as universally associated with medical student resilience [34, 35]. These attributes are consistent with Mezirow’s Transformative Learning Theory which describes how adults experiencing a transformative learning journey can see themselves differently when they have autonomy and the ability to be self-aware and to critically reflect on action [36]. Students change their frame of reference from dependent students who expect to know the right answer, to semi-autonomous novice clinicians who can accept clinical uncertainty and work without being distressed by their limitations [37].

LIC students describe a constructivist learning process where they were responsible for creating their own meaning of doctoring through experiencing clinical encounters and reflecting on these experiences [38]. *Staying on course* may be initially perceived in terms of getting through the syllabus. When viewed through a social learning theory lens, medical students are not solely engaged in “learning to do medicine”, but rather they are “learning to be doctors”. LICs optimise opportunities for medical students to be agentic learners as they actively move their focus from textbook knowledge to patient-orientated clinical engagement [39]. During this challenging process students move from naïve states of unconscious incompetence to unconscious competence as they face their own limitations [40]. As novice team members, with a legitimate role in patient care, students incrementally become consciously competent in providing care for individuals with common and important conditions [41]. Learning required for independent performance as a clinician is developed over time in LICs through ‘collaborative thinking and acting between the expert and novice’ known as proximal guidance [42]. Clinician supervisors have an important role in keeping students on course as they navigate their profound learning journey. Proximal guidance enhances resilience as students simultaneously experience being challenged and encouraged.

### Limitations

The limitations of this study include the small sample size and recruitment from one university LIC program only. Another limitation is the reliance on the students’ self-reported level of resilience, and on the staff and educators experience in assessing stress and resilience in students. A longitudinal multi school study with a larger sample size is recommended for future research. Assessment of stress and resilience using validated tools at the beginning and end of the academic year would also be useful.

## Conclusion

The Flinders model of student resilience contests the notion that medical courses can ensure student wellbeing solely through the provision of additional stand-alone emotional and tangible supports without a clear understanding of how the course influences student resilience. This model highlights that resilience is dependent on the strength of all three components of the model, just as a triangle provides inherent strength within a bridge. As students embark on their first clinical year of training, a LIC can build resilience in students through continuity of relationships, providing guidance in their learning and reinforcing their personal growth.
